# Unveiling the pathological mosaic of phyllodes tumors

**DOI:** 10.11604/pamj.2025.52.177.40475

**Published:** 2025-12-23

**Authors:** Suhit Naseri, Samarth Shukla

**Affiliations:** 1 Department of Pathology, Datta Meghe Medical College, Datta Meghe Institute of Higher Education and Research, Wanadongri, Nagpur, India

**Keywords:** Phyllodes tumors, breast neoplasms, malignant potential

## Image in medicine

Phyllodes tumors are rare fibroepithelial neoplasms of the breast, accounting for approximately 1% of all breast tumors, and are characterized by a distinctive leaf-like architectural pattern from which their name is derived. They commonly present as a painless, slowly enlarging breast mass, most often affecting women in the third to fifth decades of life. Clinically, these tumors may show prolonged indolent growth followed by a phase of rapid enlargement, raising suspicion for malignancy. A 36-year-old female presented with a painless lump in the left breast for 3 years, with a gradual increase in size over the preceding 3 months. Gross examination revealed multiple irregular fibrofatty tissue fragments aggregating to 8 x 7 x 3 cm, with a lobulated appearance. Histopathological examination revealed a biphasic fibroepithelial lesion with a characteristic leaf-like architecture at low magnification (H&E, 4x). The tumor was composed of epithelial-lined clefts supported by an underlying stromal component (H&E, 10x). On higher magnification, the stromal cells showed mild cellularity with minimal nuclear atypia and no significant mitotic activity (H&E, 40x). The patient underwent complete surgical excision with adequate margins. The postoperative course was uneventful, and short-term follow-up showed no evidence of recurrence. Understanding the pathological features and behavior of these rare tumors is essential for providing optimal care to patients affected by phyllodes tumors and ensuring favorable outcomes.

**Figure 1:**
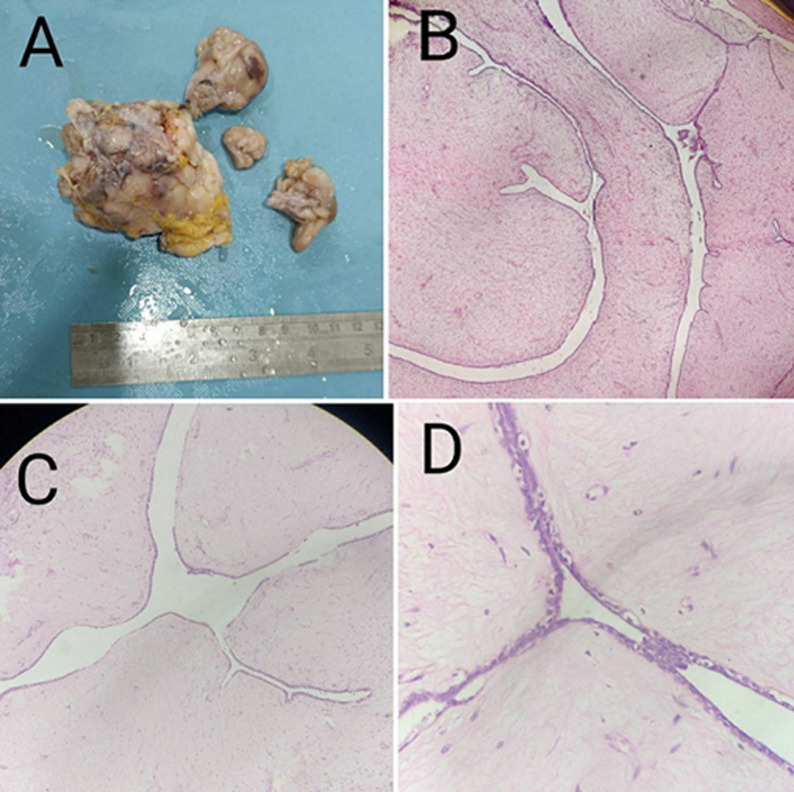
A) gross examination revealed multiple irregular fibrofatty tissue fragments aggregating to 8×7×3cm, with a lobulated appearance; B) histopathological examination demonstrated a biphasic fibroepithelial lesion with a characteristic leaf-like architecture at low magnification (H&E, 4×); C) the tumor was composed of epithelial-lined clefts supported by an underlying stromal component (H&E, 10×); D) on higher magnification, the stromal cells showed mild cellularity with minimal nuclear atypia and no significant mitotic activity (H&E, 40×)

